# Immobilization of Active Antibodies at Polymer Melt Surfaces during Injection Molding

**DOI:** 10.3390/polym14204426

**Published:** 2022-10-20

**Authors:** Thor Christian Hobæk, Henrik J. Pranov, Niels B. Larsen

**Affiliations:** 1 Department of Health Technology, DTU Health Tech, Technical University of Denmark, Ørsteds Plads 345C, 2800 Kongens Lyngby, Denmark; 2 Inmold A/S, Savsvinget 4B, 2970 Hørsholm, Denmark

**Keywords:** thermoplastic polymers, injection molding, protein transfer, antibody, ELISA, AFM, XPS

## Abstract

We demonstrate the transfer and immobilization of active antibodies from a low surface- energy mold surface to thermoplastic replica surfaces using injection molding, and we investigate the process at molecular scale. The transfer process is highly efficient, as verified by atomic force microscopy (AFM) and X-ray photoelectron spectroscopy (XPS) of the mold and replica surfaces. AFM analysis reveals partial nanometer-scale embedding of the protein into the polymer matrix as a possible mechanism of permanent immobilization. Replicas with rabbit anti-mouse IgG immobilized as capture antibody at the hot polymer melt surface during injection molding show similar affinity for their antigen (mouse IgG) in sandwich enzyme-linked immunosorbent assay (ELISA) as capture antibodies deposited by passive adsorption onto a bare thermoplastic replica. The transferred antibodies retain their functionality after incubation in serum-containing cell medium for >1 week. A mold coating time of 10 min prior to injection molding is sufficient for producing highly sensitive ELISA assays, thus enabling the short processing cycle times required for mass production of single-use biodevices relying on active immobilized antibodies.

## 1. Introduction

Immobilization of proteins onto solid substrates is of great importance in many applications including protein microarrays for proteomics [1], development of personalized drugs [2] and point-of-care diagnostics [3], immunoassays such as the enzyme-linked immunosorbent assay (ELISA) [4], and sorting of cells through surface antigen-recognition [5,6]. Most existing biochips employing covalent protein immobilization are made from glass or silicon (oxide) that can be modified by silane-based chemistry [7,8]. However, there is great interest in using industrially moldable polymers for the production of biochips and miniaturized lab-on-a-chip devices due to lower material cost, faster production, and reduced processing cost [9]. This is especially important for clinical applications that require disposable devices that eliminate the risk of sample contamination [10]. Injection molding has for decades been the most industrially relevant method for shaping polymers, and it is also increasingly used as production technology for microfluidic devices [11]. It is a rapid replication technique with cycle times from 1–30 s, which involves heating of a thermoplastic polymer to substantially above its glass transition temperature (*T_g_*), typically >250 °C, followed by injection of the polymer melt into a closed mold maintained below *T_g_*, where the polymer solidifies in its final shape.

Popular thermoplastic materials for life science applications include polystyrene (PS), polypropylene (PP), polycarbonate (PC), poly(methyl methacrylate) (PMMA), and cyclic olefin copolymers (COC). These are, however, chemically inert in their native form [12]. Covalent immobilization of proteins therefore requires prior chemical modification to render the polymer surface reactive towards functional groups on exposed amino acids. Activation of plastic surfaces has previously been demonstrated by plasma treatment, oxidation using a base, or by UV irradiation, followed by introduction of amine groups [13,14,15,16], carboxylic acid groups [17], aldehydes [18,19], *N*-hydroxysuccinimide (NHS) esters [20], and epoxides [21]. We have also previously demonstrated photo-immobilization of antibodies on a polyethylene glycol (PEG)coating with low non-specific protein adsorption bound on a native PS surface [22,23]. Although a high protein density and, in some cases, control of the orientation of immobilized antibodies could be achieved with these methods, they all involve wet chemical processing steps after polymer shaping, which inevitably increase production time and cost.

An untraditional approach for antibody immobilization is the direct entrapment of the protein on a polymer surface during molding, where pre-adsorbed proteins on a mold surface are transferred to the polymer replica during shaping. This has been demonstrated with poly(dimethylsiloxane) (PDMS) [24,25], which in liquid form is cast on an antibody-coated poly(tetrafluoroethylene) (PTFE) mold followed by thermal curing and separation of the replica. PDMS is the preferred prototyping material for lab-on-a-chip devices [26], and is based on a simple molding process. However, it has numerous limitations compared to thermoplastics, such as high bulk absorption of hydrophobic compounds from solution [27], leaching of uncrosslinked polymer chains into solution [28], hydrophobic recovery [29], and very long curing times (of hours to days) at elevated temperatures.

We have previously reported on a “dry” approach based on fast injection molding of PMMA and PP to achieve simultaneous topography replication and pattern transfer of horseradish peroxidase (HRP), fibronectin, and avidin from the mold to the replica surface, with some retained biochemical activity [30]. The process requires physical contact between the hot polymer melt and the proteins on the mold surface at thermal conditions which are known to denature proteins at sub-second time scales. However, the cooling rate at the mold/melt interface was predicted to be sufficiently fast (ns—µs time scale) to prevent complete denaturation. With this method, no prior functionalization of the polymer replicas is required, allowing for complete process cycle times to be reduced to 30 s in a fully automated setup.

We also explored antibody transfer and immobilization in our former report but without achieving biological recognition: immobilized antibodies could function as antigens for antibodies added in solution, but the immobilized antibodies could not recognize dissolved antigens. Here, we demonstrate the direct transfer of functionally active capture antibodies, specifically anti-mouse IgG, from a low surface-energy fluorocarbon-coated mold surface to a COC replica by injection molding, avoiding the need for chemical modification of the antibodies and avoiding wet chemical post-processing. In addition, we present a quantitative chemical and structural analysis of the highly efficient transfer procedure at the nanometric-length scale, as well as a biofunctional analysis showing that the antigen-binding ability of the transferred antibodies is retained at a level equal to that of antibodies deposited on the polymer surface by conventional passive adsorption.

## 2. Materials and Methods

### 2.1. Materials

Dulbecco’s Phosphate Buffered Saline (dPBS, D8537), Dulbecco’s Modified Eagle’s Medium (DMEM, D5030), IgG from mouse serum (I5381), anti-mouse IgG (whole molecule) antibody produced in rabbit (M7023), essentially IgG-free bovine serum albumin (BSA; ≤25 ng IgG/mg BSA, A9085), fetal bovine serum (FBS, F2442), penicillin/streptomycin (P/S, P4333), and 3,3′,5,5′-tetramethylbenzidine (TMB) liquid substrate system for ELISA (T0440) were purchased from Sigma Aldrich (St. Louis, MO, USA). Rabbit anti-mouse IgG antibody conjugated with horseradish peroxidase (HRP) (P0260) was purchased from Dako (Glostrup, Denmark). All water used was purified with a Millipore MilliQ system (Boston, MA, USA). The blocking buffer consisted of 0.05 % *v/v* Tween 20 (Merck-Schuchardt, Hohenbrunn, Germany) and 0.1% *w/v* BSA in 1 × dPBS. The wash buffer consisted of 0.05% *v/v* Tween 20 in 1 × dPBS. The colorimetric reaction was stopped by addition of 0.5 M H_2_SO_4_ in MilliQ water. Protein stability tests used an incubation buffer of 10% *v/v* FBS and 100 U/mL (P/S) in DMEM. All incubation steps were conducted at room temperature (25 °C) unless otherwise stated. From ibidi (Martinsried, Munich, Germany) were purchased 12-well removable silicone chambers (81201) and 8-well sticky-slide chambers (80828). Nunc 96-well polystyrene plates (260860) were acquired from Fischer Scientific (Roskilde, Denmark). For injection molding, TOPAS^®^ 8007-S04 (COC) was purchased from Topas Advanced Polymers (Frankfurt-Höchst, Germany) and shims of electroplated nickel (300 µm thickness) were obtained from DVD Norden (Sakskøbing, Denmark).

### 2.2. Surface Modification of Nickel Mold Inlays

Mold inlays in the form of electroplated nickel shims were used as mold substrates for the protein transfer. To improve protein transfer, a tri-layered low surface-energy fluorocarbon-based coating was applied on the surface of the inlays by initial atomic layer deposition (ALD, PICOSUN R-150, Picosun Oy, Espoo, Finland) of 5 nm Al_2_O_3_ and 5 nm SiO_2_, followed by molecular vapor deposition (MVD, MVD 100, Applied Microsystems Inc., San Jose, CA, USA) of a monolayer of heptadecafluoro-1,1,2,2-tetrahydrodecyltrichlorosilane (FDTS) using a previously described process [31]. The mold-inlay surfaces were cleaned between each protein transfer cycle by plasma treatment (200 sccm O_2_, 250 W, 300 s; MVD 100, Applied Microsystems Inc., San Jose, CA, USA) and subsequent reapplication of the FDTS-coating using MVD, unless otherwise stated.

### 2.3. Transfer of Capture Antibodies during Injection Molding

Rabbit anti-mouse IgG was deposited by passive adsorption onto the FDTS-coated mold inlays by dispensing a solution of 10 µg/mL protein in dPBS for 10 or 60 min as specified. Excess protein and salt from the buffer were removed by immersing the molds in 30 mL MilliQ water for 1 min, before the mold-inlay surfaces were dried with a pressurized air gun. The protein-coated inlays were immediately installed in a commercial injection molding machine (VC 80/45, Engel, Schwertberg, Austria). Molten COC, initially at a temperature of 250 °C, was subsequently injected into the mold cavity that was maintained at a constant temperature of 20 °C, with a volumetric injection rate of 43 cm^3^/s. The replicas with transferred protein were kept in a sealed zip-lock bag at 4 °C until analysis by ELISA.

### 2.4. Characterization of Transfer Efficiency by Surface Analysis

The mold-inlay surface before protein deposition, after protein deposition, and after protein transfer by injection molding, as well as the surface of the polymer replicas with and without transferred proteins, were characterized by atomic force microscopy (AFM, XE-100, Park Systems, Suwon, Korea) using BudgetSensor-300 cantilevers operated in intermittent contact mode. The recorded micrographs were adjusted with a plane fit at an average zero height. X-ray photoelectron spectroscopy (XPS) was performed with a K-Alpha spectrometer (Thermo Scientific, East Grinstead, UK) using a 400 µm wide monochromatized Al K_α_ X-ray beam spot and analyzer pass energies of 50 and 200 eV for high resolution and survey spectra, respectively. Elemental composition analysis and deconvolution of C1s spectra were performed using the instrument manufacturer’s Avantage software package. Contact angles of water, diiodomethane, and benzyl alcohol on the mold inlay and COC replica surfaces were measured optically using an OCA 20 system (dataphysics, Filderstadt, Germany). The surface energy with dispersive and polar components was calculated using the Owens-Wendt method [32]. The advancing and receding contact angles were determined by the dynamic sessile drop method, through adding/removing liquid at 0.1 µL/s and calculating the average angle while the length of the contact line increased/decreased. At least 5 drops on different positions on each sample were measured.

### 2.5. ELISA with Injection Mold Transferred Capture Antibody

A sandwich ELISA using the injection molded anti-mouse IgG antibody as capture antibody was used to assess the functionality of the immobilized protein. A 12-well silicone chamber was mounted on the polymer replica slides with transferred antibody before adding 300 µL of blocking buffer to each well and incubating for 1 h. The excess solution was tapped out and 100 µL mouse IgG (antigen) in blocking buffer was added and incubated for 16 h at 4 °C. Each well was washed for 6 × 3 min with washing buffer, and 100 µL 0.5 µg/mL rabbit anti-mouse IgG-HRP (detection antibody) in blocking buffer was added and incubated for 1 h. Afterwards, the same washing procedure was repeated followed by adding 100 µL TMB substrate and incubating for 10 min on a shaking table. 100 µL 0.5 M aqueous H_2_SO_4_ was added to stop the enzymatic reaction, and 100 µL supernatant was transferred from each well on the polymer replicas onto a transparent 96 well PS plate. The absorbance was read at 450 nm using a Victor3 plate reader (Perkin Elmer, Santa Clara, USA). For comparison, capture antibody was deposited on uncoated polymer replica slides by passive adsorption from solution, using the same coating procedure and concentrations as for the mold inlays, before washing with MilliQ water, drying, and mounting the silicone well chambers. As a negative control, polymer replica slides without capture antibody were analyzed using the same assay.

For the protein stability tests, a bottomless 8-well slide was attached to the polymer replicas by adhesive tape, and 500 µL of DMEM with 10% *v/v* FBS and 100 U/mL P/S was added. The slides were incubated in a sealed bag for 9 days at 4 °C to minimize the risk of infection, before performing a sandwich ELISA as described. It should be noted that the slides with transferred proteins were stored in the dry state for 3 weeks at 4 °C before the stability tests were initiated.

### 2.6. Data Analysis and Curve Fitting

Quantitative data are reported as mean ± standard error of the mean (SEM) unless otherwise stated. Statistical significance was evaluated using Welch’s t-test for two samples having possibly unequal variances [33].

The antigen standard curves were quantified using a Four Parameter Logistic (4PL) curve fit, $A=A_{0}+\left(A_{max}-A_{0}\right)/\left(1+{\left(\frac{c}{EC_{50}}\right)}^{\alpha }\right)$, where A is the measured absorbance, $A_{0}$ the absorbance at zero analyte concentration, $A_{max}$ the saturation absorbance from the analyte, c the analyte concentration, $EC_{50}$ the half-maximum effective concentration, and α is the Hill curve steepness [34]. A limit of detection for each assay was defined as the mean + 3× the standard deviation of the zero antigen well [35].

## 3. Results and Discussion

### 3.1. IgG Is Transferred with High Efficiency from the Mold Inlay to the Polymer Replica

Efficient transfer of antibodies during the injection molding process is essential to produce replicas with biofunctionally active surfaces. Initial experiments compared IgG transfer from mold inlays of native hydrophilic nickel to inlays of nickel with a low surface-energy coating presenting a molecular layer of fluorocarbon-silane (FDTS) at the surface. Native nickel inlays resulted in markedly poorer transfer efficiency (data not shown). Thus, only FDTS-coated mold inlays were considered in the further work. Experiments were performed by initial protein coating of the mold inlay from solution, followed by insertion of the inlay into the mold cavity, and final injection molding to produce the polymer replica. The transfer efficiency was evaluated qualitatively by atomic force microscopy (AFM) and contact angle analysis and quantitatively by X-ray photoelectron spectroscopy (XPS) on both the mold-inlay and replica surfaces.

**Figure 1:**
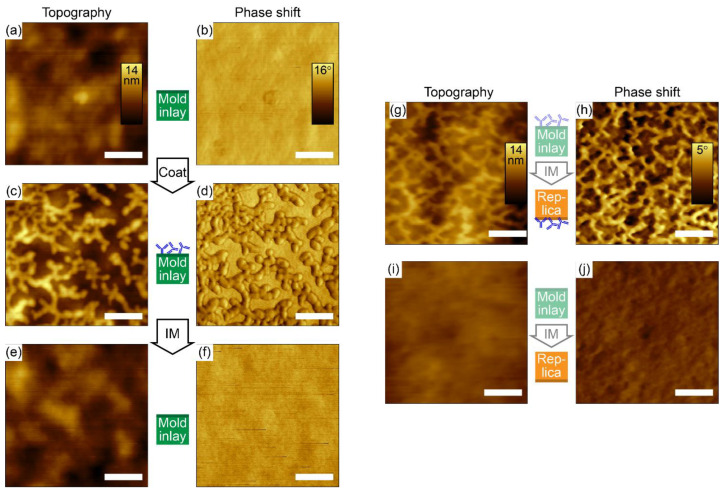
Adsorbed protein molecules are efficiently transferred to polymer replicas during injection molding, as visualized by AFM topography and phase shift micrographs of the mold-inlay surface and of the polymer replica surface with or without protein (IgG) coating. (**a**,**b**) Mold inlay prior to IgG coating. (**c**,**d**) Mold inlay after IgG coating. (**e**,**f**) Mold inlay after injection molding. (**g**,**h**) COC replica injection molded using an IgG-coated mold inlay. (**i**,**j**) COC replica injection molded using an uncoated mold inlay. The color scale corresponds to 14 nm in all topography micrographs, to 16° phase shift in the mold inlay micrographs (**b**,**d**,**f**), and to 5° phase shift in the replica micrographs (**h**,**j**). Scale bars are 200 nm.

Figure 1a–f shows AFM micrographs of the mold inlay surface before protein coating, after protein coating, and after protein transfer during injection. The mold inlay shows some inherent nanometer-scale roughness on length scales of hundreds of nanometers (Figure 1a,b), observed after ALD deposition of Al_2_O_3_ and SiO_2_ thin films on the nickel support to promote subsequent covalent coupling of gas-phase FDTS. The phase shift image is featureless, as expected for a chemically and mechanically homogeneous surface. Incubation of the mold inlay in dissolved IgG for 60 min results in adsorption of the proteins (Figure 1c,d), which appears in the micrographs as surface protrusions (bright dots and lines) of apparent width < 50 nm, distinctly different from the inherent roughness of the inlay. The patterns of dried antibodies on the hydrophobic fluorocarbon coated mold surface are fully consistent with the antibody patterns on hydrophobic methylated silicon earlier visualized by AFM, both in overall topology and in measured heights [36]. Injection molding using molten COC on the IgG-coated mold inlay apparently results in complete removal of protein from the mold surface, as shown in Figure 1d,e, which are visually indistinguishable from the micrographs recorded prior to IgG coating. Figure 1g–j compare polymer replicas molded against mold inlays with or without adsorbed protein. Surface structures on the replica injection molded on an IgG-coated mold inlay (Figure 1g,h) are highly similar in appearance to the protein patterns observed on the mold inlay (Figure 1c,d), which is most prominently observed in the phase shift micrograph. In contrast, the surface of replicas injection molded on uncoated mold inlays has a very low surface roughness without distinguishing features on the nanometer- to micrometer- length scale (Figure 1i,j). All topography micrographs in Figure 1 use the same color scale, and visually the transferred protein appears less protruding than on the coated mold inlay. A quantitative analysis of the height variations (Supporting Information, Figure S1) shows mean maximum heights of 5.3 nm and 3.6 nm on the mold-inlay and COC replica surfaces, respectively. The apparent height reduction of the transferred proteins is possibly caused by partial embedding into the polymer melt during injection molding, which may act as a key mechanism for stable immobilization of the transferred antibodies.

XPS was employed to obtain quantitative information on the amount of protein transferred. The elemental composition was determined (i) on the mold surface before and after deposition of IgG, (ii) on the mold surface after injection molding, and (iii) on polymer replicas resulting from molds with and without IgG transferred during injection molding transferred IgG (Table 1). The analysis showed an increase in carbon (21.4 vs. 11.4 atom%) and nitrogen (2.8 vs. <0.3 atom%) surface concentrations after IgG adsorption on the mold, as expected from the polyamide backbone of the proteins. Previous work using XPS to quantify the amount of protein adsorbed on non-nitrogen-containing surfaces showed the equivalence of 2.3 atom% nitrogen to 0.16 µg/cm^2^ antibodies, as determined by radioisotope labeling [37]. Thus, at least 0.16 µg/cm^2^ IgG adsorbs to the mold surface in the present study compared to the surface density of a full IgG monolayer at ~0.46 µg/cm^2^ reported using quartz crystal microbalance analysis [38]. After injection molding, the chemical composition of the mold surface is close to the initial values, with no detectable nitrogen (<0.3 atom%), a minor increase in the surface concentrations of carbon and fluorine, and a small decrease in the amount of oxygen. The signals from oxygen, fluorine, and silicon likely originate from the FDTS monolayer and the underlying SiO_2_ adhesion-promoting layer on the mold-inlay surface. Correspondingly, nitrogen and oxygen signals were detected on a COC polymer replica molded on an IgG-coated inlay but not on a COC (hydrocarbon) replica molded on an uncoated inlay. Similar nitrogen surface concentrations were detected on an IgG-coated mold inlay (2.8 atom%) and on a polymer replica (3.6 atom%) in support of highly efficient protein transfer from the FDTS-coated inlay.

**Figure 2:**
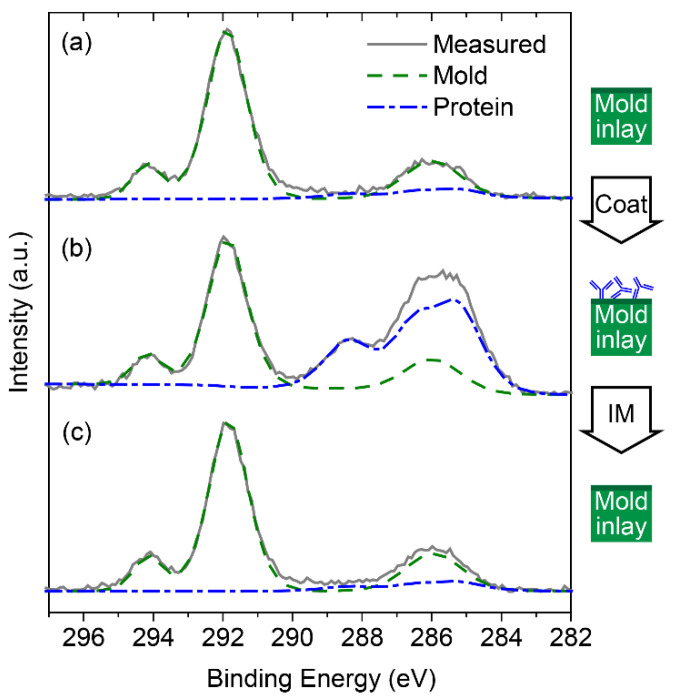
C_1s_ high-resolution XPS spectra support complete transfer of protein from the mold inlay during injection molding. (**a**) Mold-inlay surface (FDTS on SiO_2_) without proteins, (**b**) after protein deposition (“Coat”), and (**c**) after protein transfer to the polymer replica by injection molding (“IM”). The photoelectron contributions from the mold-inlay FDTS layer and the protein coating are fitted by the dashed and dash-dotted curves, respectively.

High-resolution XPS analysis of the carbon signals from the mold-inlay surfaces corroborated the results of the elemental analysis (Figure 2). Peak contributions from the mold-inlay surface components and from the adsorbed protein were extracted by curve fitting and subsequently fitted in combination to C_1s_ spectra from the mold-inlay surface at different process stages. Details of the peak fitting procedure are presented in the supporting information (Figures S2 and S3). In brief, mold-inlay peak contributions to the carbon spectra are from the fluorocarbon part of the FDTS monolayer at 294.2 eV (C*F_3_-) and 291.9 eV (-C*F_2_-) [39] and from the two methylene units closest to the SiO_2_ layer at 286.3 eV (-CH_2_-C*H_2_-CF_2_-) and 285.6 eV (-Si-C*H_2_-CH_2_-), respectively. Adsorbed proteins are assigned to peaks from the amide carbon (-NH-CR-C*(=O)-) in the protein backbone (288.4 eV), the nitrogen-bound α-carbon (-NH-C*R-C(=O)-) in the backbone as well as oxygen- and nitrogen-bonded carbon in the side groups (286.6 eV), and carbon-carbon bonds in the side groups (285.2 eV). A strong increase in the protein-associated peaks is evident after protein deposition, while contributions from these peaks are hardly detectable for the mold-inlay surface after protein transfer.

The transfer efficiency was additionally evaluated by contact-angle measurements of water droplets on the mold inlays (Figure S4). The bare surface, i.e., FDTS-coated SiO_2_, exhibited mean advancing and receding contact angles of 110.8 ± 0.6° and 96.2 ± 1.0°, respectively. Additional contact-angle analysis using diiodomethane and benzyl alcohol were performed to estimate the polar and dispersive components of the surface energy. The total surface energy was 10.1 ± 0.1 mJ/m^2^, with a polar component of 0.9 ± 0.1 mJ/m^2^ and a dispersive component of 9.2 ± 0.2 mJ/m^2^. Advancing water contact angle on the mold inlay after one injection molding cycle did not change significantly, while the receding contact angle was significantly reduced (*p* < 0.002). The mold inlay fully returned to its original state before injection molding through oxygen plasma treatment and re-coating of the inlay surface with FDTS, as shown by both the advancing and receding contact angles returning to their original values.

AFM and XPS revealed changes in topography and elemental composition associated with the presence of proteins on the surface of the polymer replica. At the same time, there was no detectable difference using either analysis method on the mold inlay before protein deposition and after injection molding. However, we observed a small, significant decrease in the receding contact angle and a corresponding increase in the contact angle hysteresis on the mold after one injection mold cycle (Figure S4). Heterogeneity in either topography or surface energy is well-known to cause hysteresis, with the hydrophilic surface species dictating the receding angle [40]. Introduction of hydrophilic groups by adsorbed proteins could therefore explain the apparent increase in contact angle hysteresis [41]. The surface properties could be completely restored by plasma oxidation and subsequent vapor phase deposition of FDTS, showing that the molds inlays can be re-used after multiple subsequent protein transfers.

The results document a highly efficient transfer process that may be mediated by stronger interfacial interaction forces of the protein with the polymer than with the mold surface, as well as by partial or full embedding of the protein in the polymer melt leading to mechanical anchoring. FDTS is a highly non-polar molecule that has routinely been used as a low surface-energy coating on moving parts in microelectromechanical systems (MEMS) [42], as well as for improved separation of polymer replicas and their molding surfaces in the format of stamps for nanoimprint lithography [43,44,45,46,47] or nickel inlays for injection molding [31,48,49,50]. Adsorbed proteins on the fluorinated surface are thus expected to interact with their support mainly by weak van der Waals forces. The olefinic COC polymer is also hydrophobic in its native form. However, the measured contact angle of water was 95.7° and the total surface energy was calculated to be 40.8 mJ/cm^2^, with the dispersive component contributing to 99.5% of the total. Prior work on the transfer of proteins using microcontact-printing (µCP) concluded that successful pattern transfer depends on a lower water wettability of the printing surface than that on the printed substrate [51]. Although the difference in water contact-angle is small between the mold and the polymer compared to what is usually reported between stamp and substrate for µCP, the transfer of antibodies might be facilitated by the almost four times lower surface energy of the FDTS-coated mold. Another explanation for the efficient transfer could be partial embedding of the proteins into the polymer during injection molding, as suggested by the lower measured height variations on the replica with transferred protein than on the protein-coated mold inlay (Figure 1 and Figure S1). Partial embedding would increase the protein-polymer interfacial area and therefore also the total interaction energy and additionally support partially mechanical anchoring of the protein in the polymer matrix, thus facilitating transfer of proteins from the low-binding mold-inlay surface.

### 3.2. IgG Immobilized during Injection Molding Retains Its Antigen-Binding Ability

**Figure 3:**
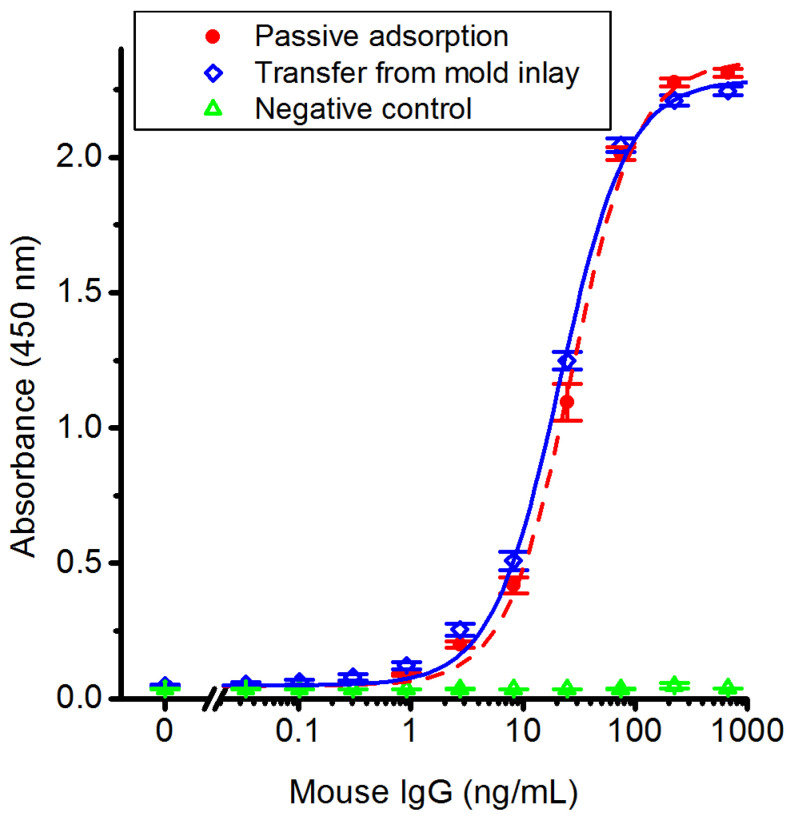
ELISA assays using antibodies immobilized during injection molding or by passive adsorption show equal sensitivity and response range. Standard curves (mouse IgG as antigen) compare the antigen-binding capacity of capture antibodies adsorbed passively to a blank polymer replica surface to those of antibodies transferred from a mold inlay. Polymer replicas without capture antibody were used as a negative control. The data points are fitted to a four-parameter logistic (4PL) curve. Error bars show the SEM (*n* = 3 for passive adsorption and negative control; *n* = 4 for transfer from mold inlays).

Antibodies are completely transferred from the mold surface to the polymer replica. We employed ELISA using an antibody as antigen to evaluate the biofunctional activity of the transferred IgG. Sandwich ELISA analysis was performed on polymer replica slides with molded antibodies and compared to replica slides with antibodies deposited by passive adsorption from PBS. The antigen-response curves, fitted with four-parameter logistic (4PL) curves in Figure 3, show no significant difference between the two methods of capture-antibody immobilization for antigen concentrations below 100 ng/mL. The linear range is from 10–75 ng/mL, and the limit of detection was 0.30 ng/mL antigen for both immobilization methods. The EC_50_ values obtained from the curve fits are 27 and 21 ng/mL antigen for passive adsorption and transfer from mold inlay, respectively.

These results show no apparent reduction of the antigen binding of the transferred capture antibodies caused by the injection molding process. This might seem surprising, as the proteins on the mold inlay are brought in contact with a polymer melt initially at 250 °C. Earlier differential scanning calorimetry (DSC) studies showed irreversible denaturation of the F_ab_ domain of IgG above 61 °C at a heating rate of 0.5 °C/min, while a higher heating rate of 5 °C/min increased the denaturation temperature to 65 °C [52]. Thus, denaturation kinetics are clearly limiting the extent of irreversible function loss. The cooling rates of the polymer melt in the immediate vicinity of the mold-inlay metal surfaces have been suggested to exceed 10^4^ °C/s [53] due to the high thermal conductivity of nickel, thereby only exposing the adsorbed protein to high temperatures for extremely short time periods. Indeed, our earlier modeling predicted that the first nanometers of polymer melt at 270 °C brought into thermal contact with a metal mold surface maintained at 30 °C are cooled to 50 °C within 100 ns [30]. Molecular dynamics simulations predicted that the unfolding time of a 61 residue α-helical protein at 225 °C is in the order of tens of nanoseconds [54], although generally the protein-folding speed limit increases with increasing number of residues in the polypeptide chain [55]. Similarly, prior experimental work demonstrated that peak temperatures of 290 °C are required to decrease the activity of horseradish peroxidase by 50% using multiple nanosecond laser pulses [56]. Antibodies transferred from the mold inlay during injection molding may therefore avoid irreversible loss of function due to heating by the polymer melt and subsequent cooling by the cold mold being on a sufficiently short timescale to avoid permanent denaturation.

### 3.3. Reduced Incubation Times Enable Industrially Relevant Production Cycle Times

**Figure 4:**
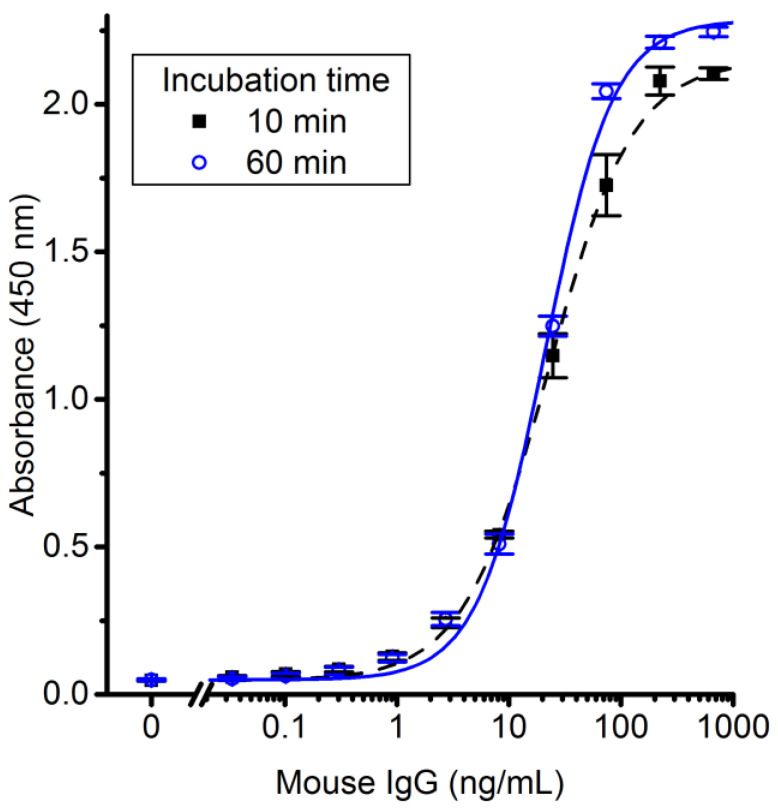
Highly sensitive ELSA assays can be produced by short mold incubation times prior to injection molding. Standard curves for mouse IgG compare the effect of incubating the capture antibody on the mold-inlay surface for 10 min and 60 min prior to transfer by injection molding. The data points are fitted by 4PL curves. Error bars show the SEM (*n* = 5 for 10 min; *n* = 4 for 60 min).

Robot replacement of a number of insertable mold parts during production is a standard process technology, but long mold-inlay incubation times would be a major challenge for viable automation and industrial application of the proposed technology, as the required number of inlays would be large to sustain low process cycle times. We targeted an injection molding cycle time of 30 s using 20 replaceable mold inlays, thus calling for an individual inlay incubation time of 10 min in comparison to 60 min incubation used for the results presented in Figure 3. Antigen-response curves using transferred antibodies with the two incubation times prior to injection molding are displayed in Figure 4. Replicas only showed significant differences in the response curves between the two incubation times for antigen concentrations ≥ 75 ng/mL (*p* = 0.04). Since the amount of adsorbed capture antibody is likely lower at the shorter incubation time, the binding of antigens to the immobilized capture antibodies is expected to saturate at lower antigen concentrations, resulting in a reduced absorbance at high antigen concentrations. Additionally, the uncertainty in the antigen response is higher for the shorter incubation time, most notably seen mostly in the upper part of the sigmoidal curve, suggesting that the density of capture antibodies is less homogeneous. However, for applications where a low analyte concentration is targeted, here in the low pM range, the shorter incubation time does not affect the assay performance.

### 3.4. Immobilized IgG Is Stable in Cell Culture Medium for Weeks

**Figure 5:**
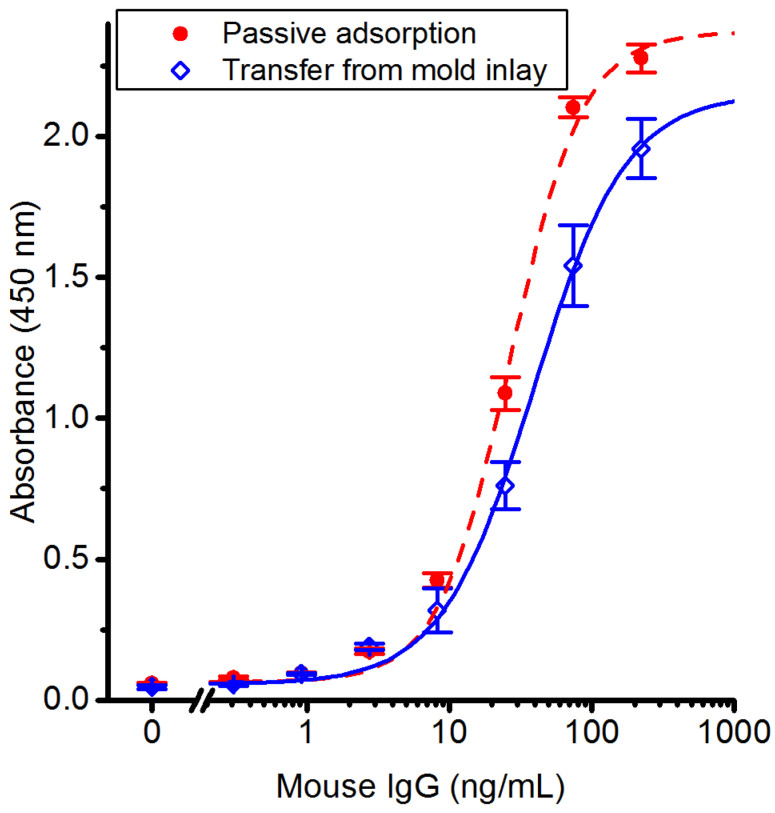
Antibodies transferred during injection molding retain their function in cell culture medium for >1 week. Standard curves are shown for mouse IgG bound to capture antibody (immobilized by passive adsorption or molded in the plastic) and stored for nine days in DMEM + 10% *v/v* FBS + 1% P/S at 4 °C. Error bars show the SEM (*n* = 3).

We investigated the stability of the transferred antibodies over time by incubating the wells in DMEM with 10% *v/v* FBS and 1% P/S for 9 days at 4 °C. The reduced temperature was chosen to minimize the risk of bacterial growth. Subsequently, we washed the wells and performed a sandwich ELISA as described in Section 2. Figure 5 shows that capture IgG transferred from mold inlays has a similar response to passively adsorbed capture IgG at antigen concentrations below 10 ng/mL and with equal limits of detection of 0.9 ng/mL. At higher antigen concentrations the response is significantly lower in magnitude (*p* < 0.05), while 4PL curve fitting yields similar EC_50_ values for both modes of capture antibody deposition (39 and 27 ng/mL for antibodies transferred during molding and passively adsorbed antibodies, respectively). It should be noted that the polymer slides with transferred capture IgG were stored in a dry state at 4 °C for 3 weeks before addition of medium, while slides with passively adsorbed capture IgG were analyzed immediately after protein deposition. The extended storage time of the transferred IgG may have reduced the protein activity.

The sensitivity of an assay for biochip applications can be improved by maximizing the surface density of the antibody and/or improving the orientation to make the antigen-recognition sites more accessible for binding [7]. The introduction of functional groups on the surface of the polymer enables covalent attachment of antibodies. Several groups have reported on the grafting of polymers with functional groups on plasma- or base-activated PMMA, such as amine-containing poly(ethyleneimine) followed by crosslinking of amines using glutaraldehyde [13,14,15] or carboxyl acid-containing poly(acrylic acid) followed by a carbodiimide crosslinker to conjugate amines on the protein [19]. The latter method has also been applied to COC [17]. Surface immobilization of proteins on COC has also been achieved by direct silanization [18] or dip-coating of a PEG-containing copolymer with epoxide [21] or NHS ester groups [20] with the added benefit of reducing non-specific adsorption. Direct coupling of proteins can also be achieved through photo-immobilization by UV illumination and a photosensitizer, which has been demonstrated for PS using different photosensitizers [22,57,58]. Although these methods have demonstrated increased sensitivity and a higher signal-to-noise ratio of ELISA, they usually contain two or more incubation steps that are time-consuming in an industrial process for high-volume manufacturing of protein biochips.

Our method is a simple one-step method of immobilizing functional antibodies on injection molded polymer slides with a retained affinity comparable to passive adsorption. With interchangeable molds, production times could be significantly lowered, with the added benefit that microchannel geometry could be shaped simultaneously by topographical structuring of the molds. Our prior work on protein transfer by injection molding demonstrated a retained functionality of avidin [30] to enable immobilization of biotinylated antibodies on pre-coated plastic plates. Direct immobilization of capture antibodies is a significant advantage due to the use of native antibodies and the omission of a wet chemical post-processing step.

The orientation of immobilized antibodies affects the accessibility of the antigen-recognition sites on the proteins. Previous studies on IgG passively adsorbed on a hydrophobic CH_3_-terminated surface suggested that initially adsorbed antibodies have a horizontal orientation (flat-on), while continued adsorption fills the interstitial spaces with more vertically oriented protein [38]. The orientation of antibodies adsorbed on the mold surface is expected to be similar to that of antibodies adsorbed on bare plastic, as only hydrophobic interactions are involved in both cases. The randomness of orientation is also likely the same after transfer to the injection molded replica. The accessibility of the binding site in the F_ab_ region of the antibody could be improved by controlling the orientation of the protein. This has previously been demonstrated using initial surface immobilization of protein A that selectively binds to the F_c_ region of the antibody, thus exposing the F_ab_ region to the surrounding buffer [14,15,19]. However, equivalent immobilization of protein A on the mold surface followed by capture-antibody incubation would result in polymer embedding of the F_ab_ region in the injection molded replica. One path to proper final orientation would be covalent immobilization of the antigen on the mold surface prior to antibody incubation, as demonstrated previously for microcontact printing of proteins [59]. Definable orientation of antibodies immobilized by injection molding might significantly improve their antigen-binding ability at the expense of higher process complexity.

## 4. Conclusions

Antibodies passively adsorbed on a surface functionalized mold-inlay surface can be transferred with a high efficiency to a COC replica slide by injection molding. Insignificant amounts of protein residuals remain on the mold surface after transfer, as verified by AFM and XPS, thus making multiple subsequent protein transfers using the same mold possible. The antigen-binding ability of the transferred capture antibody is similar to that of antibodies adsorbed by passive adsorption directly on polymer surfaces, as verified by sandwich ELISA. In addition, short incubation times (10 min) of antibody solution on the mold surface prior to transfer results in fully satisfactory antigen-response curves on replicas with transferred antibodies. Finally, antibodies transferred during injection molding retain their functionality after three weeks of dry storage followed by nine days of immersion in standard serum-containing cell culture medium. Thus, direct immobilization of antibodies at a polymer melt surface during injection molding is a promising approach for high-volume production of protein-functionalized disposable polymer biochips for immunoassays or cell-capture applications.

## Figures and Tables

**Table 1:** Elemental composition, as determined by XPS, of mold and replica surfaces with or without protein (IgG) coating before or after injection molding. The values are the average of two measurement points on each sample. The detection limit is 0.2 atom%.

	Elemental Composition (atom%)				
Sample Type	C	N	O	F	Si
Mold-inlay surface (FDTS on SiO_2_)	11.4	-	39.5	24.4	24.8
IgG-coated mold inlay	21.4	2.8	34.2	21.2	21.0
IgG-coated mold inlay after injection molding	11.9	-	38.6	24.7	24.8
Polymer replica from uncoated mold inlay	99.9	-	0.1	-	-
Polymer replica from IgG-coated mold inlay	90.7	3.6	5.3	-	-

## Data Availability

Not applicable.

## Supplementary Materials

The following supporting information can be downloaded at: https://www.mdpi.com/article/10.3390/polym14204426/s1, Figure S1: AFM height analysis of antibody coating on mold inlay and polymer replica; Figures S2 and S3: Peak fitting of the XPS C_1s_ peak of an uncoated and an antibody-coated mold inlay surface; Figure S4: Contact angle analysis of the mold inlay surface before and after transfer of antibodies.
