# Supplementary material for: Immobilization of Active Antibodies at Polymer Melt Surfaces during Injection Molding
Source: Polymers (Basel). 2022 Oct 20;14(20):4426. doi: 10.3390/polym14204426 (PMC9606872; doi:10.3390/polym14204426)
Supplement: Supplementary file 1 [file polymers-14-04426-s001.zip › polymers-1977872-supplementary.pdf]

Supporting Information for

Immobilization of active antibodies at polymer melt surfaces during injection molding

Thor Christian Hobæk[†], *Henrik J. Pranov*[‡], *Niels Bent Larsen*[*,†]

[†] Department of Health Technology, Technical University of Denmark, Ørsteds Plads 345C, 2800 Kgs. Lyngby, Denmark

[‡] Inmold A/S, Savsvinget 4B, 2970 Hørsholm, Denmark

*Correspondence to: Niels B. Larsen, e-mail: nibl@dtu.dk

Antibody surface layer thickness on coated mold inlays and on injection molded replicas

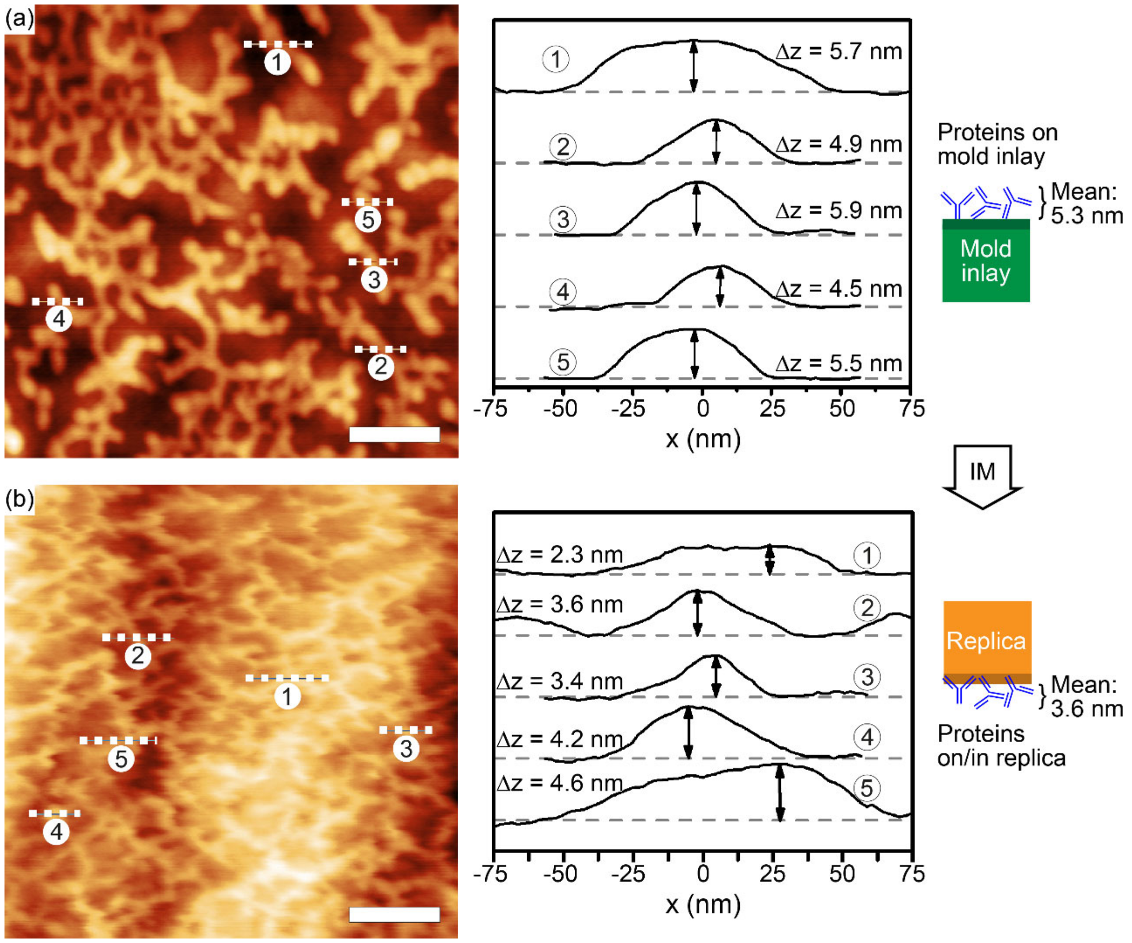

Figure S1. Atomic Force Microscopy of (a) a mold inlay coated by IgG prior to injection molding and (b) a COC replica resulting from injection molding using an IgG-coated mold inlay. The center panels show cross-sectional height profiles along the numbered lines indicated in the AFM micrographs. The mean height of protrusions on (a) the mold inlay is 5.3 nm versus 3.6 nm on (b) the COC replica, consistent with partial embedding of the IgG in the COC polymer matrix. Scale bars in the micrographs are 200 nm.

Quantitative curve fitting of the protein and mold inlay components of the XPS C1s spectra

Curve fitting proceeded by first identifying and fitting the peak pattern arising from the fluorocarbon layer of the native mold inlay (Figure S2), including the relative peak separations and intensities, followed by fitting the protein contributions on the IgG coated mold inlay (Figure S3) while only numerically scaling the complete peak pattern obtained on the uncoated mold inlay. Specifically, peaks for the fluorocarbon layer (Figure S2) were added at 285.6 eV (A), 286.3 eV (B), 291.9 eV (C) and 294.2 eV (D), where peaks A and B are ascribed to the two carbons proximal to the silicon in FDTS, peak C to $-CF_2$ groups, and peak D to $-CF_3$ groups. Peaks A and B were constrained to be separated by 0.7 eV based on the binding energy difference of methylene groups in poly(vinylidene fluoride) (neighboring $–CF_2$ groups) and poly(vinyl fluoride) (neighboring –CF groups)[1]. After fitting to the XPS measurement using these constraints, the peaks A, B, and D were further constained to be separated from peak C by -6.25 eV, -5.55 eV, and +2.31 eV, respectively. Finally, the area of peaks A, B, and D were constrained relative to peak C at C/6.6, C/6.49 and C/6.51, respectively, and the fitted full width at half maximum (FWHM) of all peaks was locked.

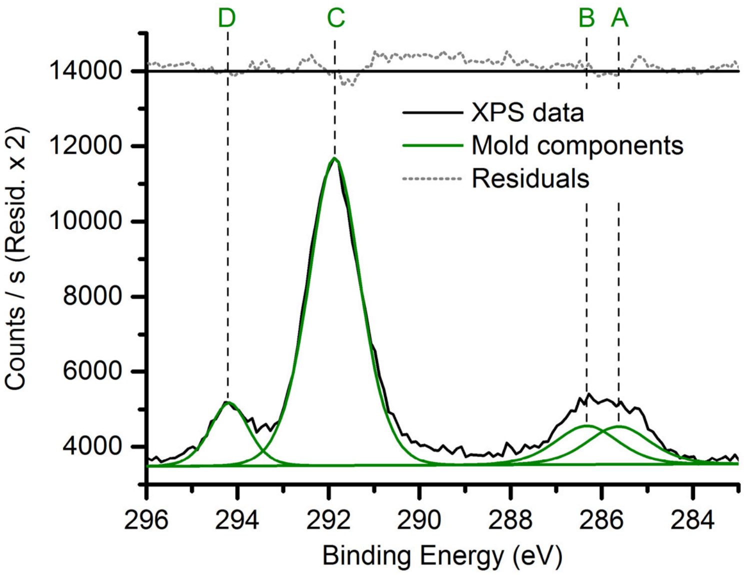

Figure S2. Peak fitting of the components of the uncoated mold inlay surface.

The peak fit parameters with all constraints were then applied on the XPS results from the IgG-coated mold inlay (Figure S3). Three new peaks were added at 285.2 eV (E), 286.5 eV (F) and 288.4 eV (G) originating from carbonyl carbons in the backbone and carboxyl carbons in the side-chains of the amino acids (C=O and COOH), the amine-bound α-carbon (C-N), and carbon single bonds (C-C), respectively. After curve fitting, peaks E, F, and G were constrained at a separation of -6.63 eV, -5.34 eV, and -3.46 eV from peak C, respectively. The area of peaks F and G were constrained relative to peak E at E/1.56 and E/1.73, respectively. The FWHM of all peaks were locked, and the data were fitted again. After assigning the parameters of all seven peak components, the constrained peak

parameters were applied again to the measured data for the uncoated mold inlay and to the IgG-coated mold inlay.

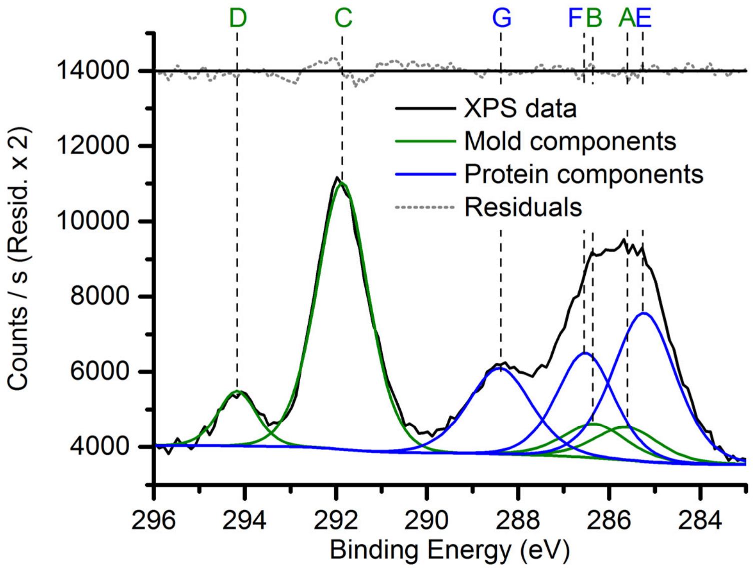

Figure S3. Simultaneous peak fitting of the mold inlay components and the protein components of the IgG-coated mold inlay surface.

Contact angle measurements on mold surface before and after transfer of IgG

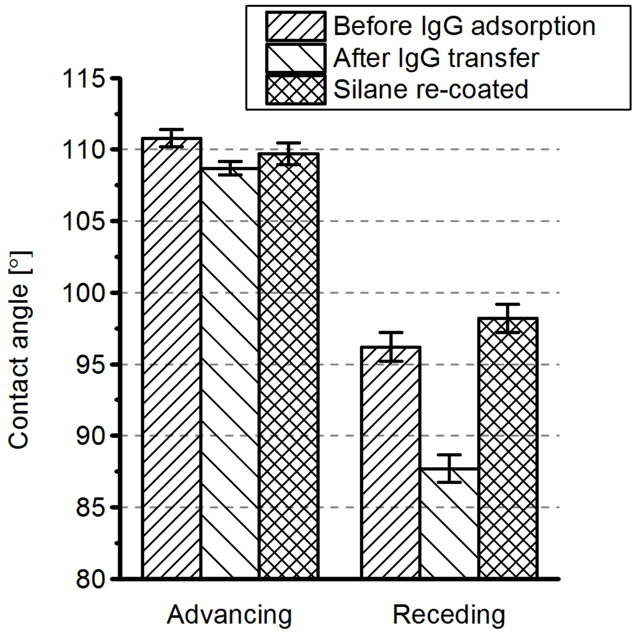

Figure S4. Advancing and receding water contact angles on mold inlay surfaces (FDTS-coated SiO_2) before adsorption of IgG, after IgG transfer to a polymer replica during injection molding, and after a new FDTS-coating is applied. Error bars show the SEM (n=3).

References

(1) Beamson, G.; Briggs, D. *High Resolution XPS of Organic Polymers - The Scienta ESCA300 Database*; John Wiley & Sons: Chichester, 1992.
